# SPICES: a particle-based molecular structure line notation and support library for mesoscopic simulation

**DOI:** 10.1186/s13321-018-0294-7

**Published:** 2018-08-09

**Authors:** Karina van den Broek, Mirco Daniel, Matthias Epple, Hubert Kuhn, Jonas Schaub, Achim Zielesny

**Affiliations:** 1 Inorganic Chemistry and Center for Nanointegration Duisburg-Essen (CeNIDE), University of Duisburg-Essen, Essen, Germany; 2 Institute for Bioinformatics and Chemoinformatics, Westphalian University of Applied Sciences, August-Schmidt-Ring 10, 45665 Recklinghausen, Germany; 3 CAM-D Technologies, Solingen, Germany

**Keywords:** Molecular structure representation, Line notation, Mesoscopic simulation, Dissipative Particle Dynamics, DPD

## Abstract

Simplified Particle Input ConnEction Specification (SPICES) is a particle-based molecular structure representation derived from straightforward simplifications of the atom-based SMILES line notation. It aims at supporting tedious and error-prone molecular structure definitions for particle-based mesoscopic simulation techniques like Dissipative Particle Dynamics by allowing for an interplay of different molecular encoding levels that range from topological line notations and corresponding particle-graph visualizations to 3D structures with support of their spatial mapping into a simulation box. An open Java library for SPICES structure handling and mesoscopic simulation support in combination with an open Java Graphical User Interface viewer application for visual topological inspection of SPICES definitions are provided.
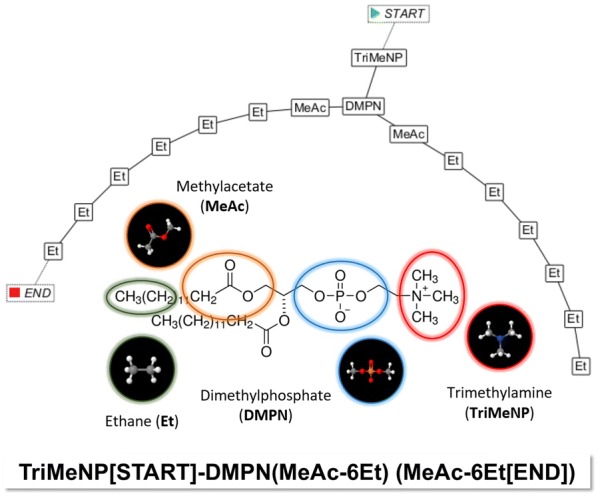

## Background

A molecular simulation task comprises three successive steps: The definition of a simulation job with all necessary input information (preparation step), the actual loop over discrete integration time steps to numerically solve the equations of motion (the actual simulation step) and the analysis of the simulation record with all calculated results (evaluation step). The first (preparation) step of this triad has to provide data structures that can be leveraged by the algorithms of the second (simulation) step in an optimized manner to allow for a maximum performance of their interplay. This is commonly achieved by definition of adequate sets of arrays that encode all necessary molecular information like spatial positions or bonds of the interacting entities. The content of these arrays is usually provided by large tabular ASCII files that are often (at least partly) edited by hand. An example of these ASCII files may be found at [1] for 1,2-Dimyristoyl-sn-glycero-3-phosphocholine (DMPC) phospholipid molecules of a bilayer-membrane simulation task where each line contains an interacting entity, its spatial x,y and z coordinates, line offsets to bonded entities and specific indices for additional force assignments. The manual creation of these machine-oriented contents is not only a tedious but an error-prone type of work: For all but the simplest molecular ensembles errors are likely to be generated that may spoil the whole simulation process. Thus there is a valid necessity to prevent mistakes by safeguarded operations and to reduce manual preparation overhead by adequate automation.

**Fig. 1:**
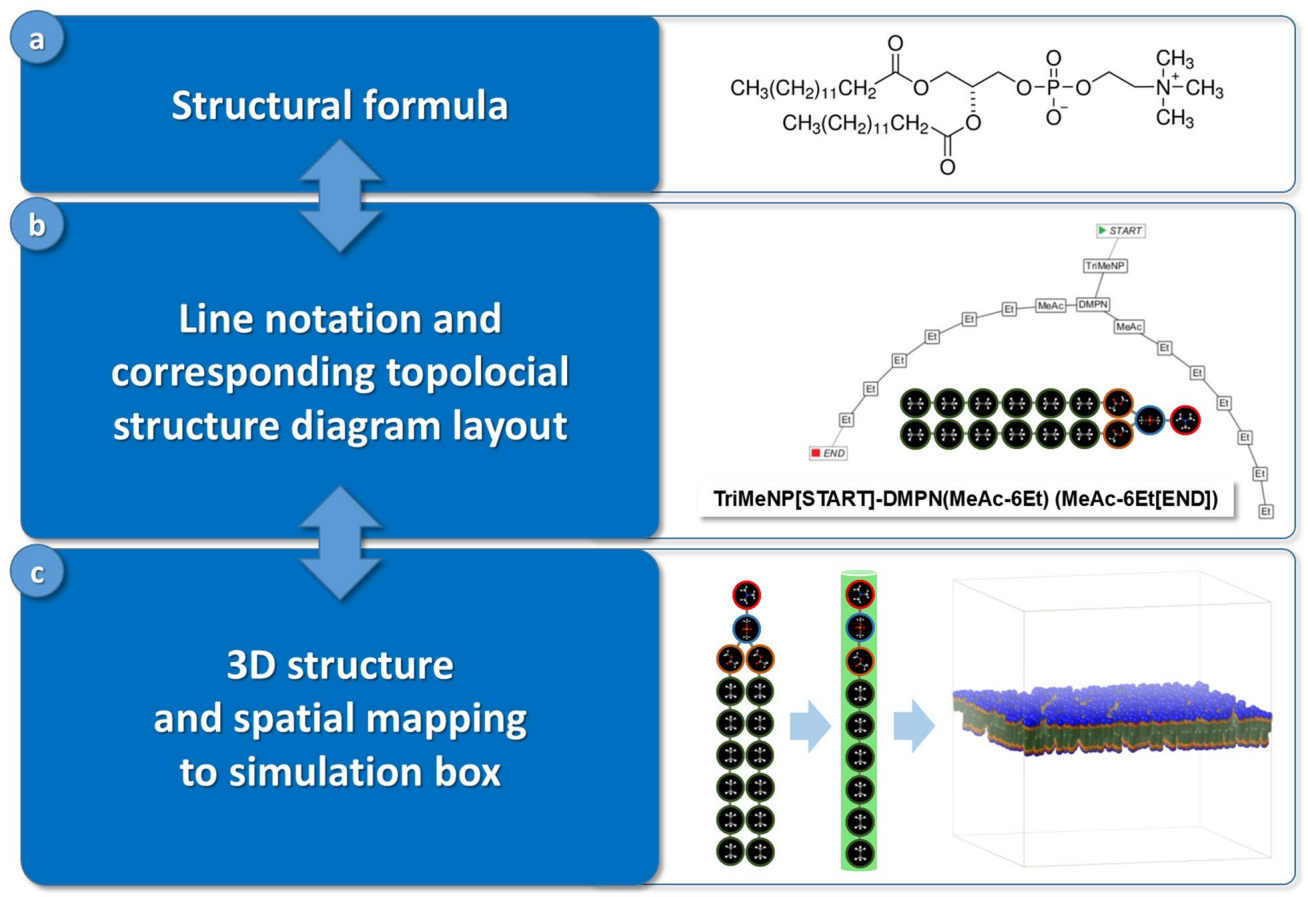
Interplay between different encoding levels of molecular structures for a preparation step of a molecular simulation task (with examples of this work, compare Figs. 2, 4 and 5). **a** Structural formula of a DMPC phospholipid. **b** SPICES line notation of the particle-based topological DMPC structure with its corresponding structure diagram layout/particle graph and illustration of the particle bonds. **c** Conversion of the topological particle structure to a compressed 3D tube geometry plus spatial mapping into an oriented bilayer compartment of the simulation box

Cheminformatics aims at supporting efficient and errorless human–machine interfaces where adequate molecular structure representations (line notations, connection tables, XYZ tables or Z-matrices, fragment codes or fingerprints, file formats like MOL file or PDB file) are at heart of the discipline [2]. The majority of existing structure representations are atom-based descriptions that comprise characteristic properties and topological or spatial aspects concerning a molecule’s atomic composition [2, 3] with additional approaches towards fragment-based molecular representations especially for polymers [4–8]. In order to support the preparation step of a molecular simulation task cheminformatics methods allow for an effective interplay of different levels of molecular encoding that are constitutive for a comfortable and safe human–machine interface (see Fig. 1): The topological structural formula is a common way used by molecular scientists to represent a chemical compound (e.g. drawn by hand with a structure editor or manually selected from structure repositories). Alternatively the compound may be represented by a textual line notation—where the interplay between structural formula and line notation may be realized by mutual conversion methods like an adequate structure diagram layout. The following transition from topological representations to 3D structures allows for the final mapping to their spatial positions within a simulation box which completes the preparation step. All prepared information may then be stored in form of the tabular ASCII files sketched above as an input for the actual simulation step.

**Fig. 2:**
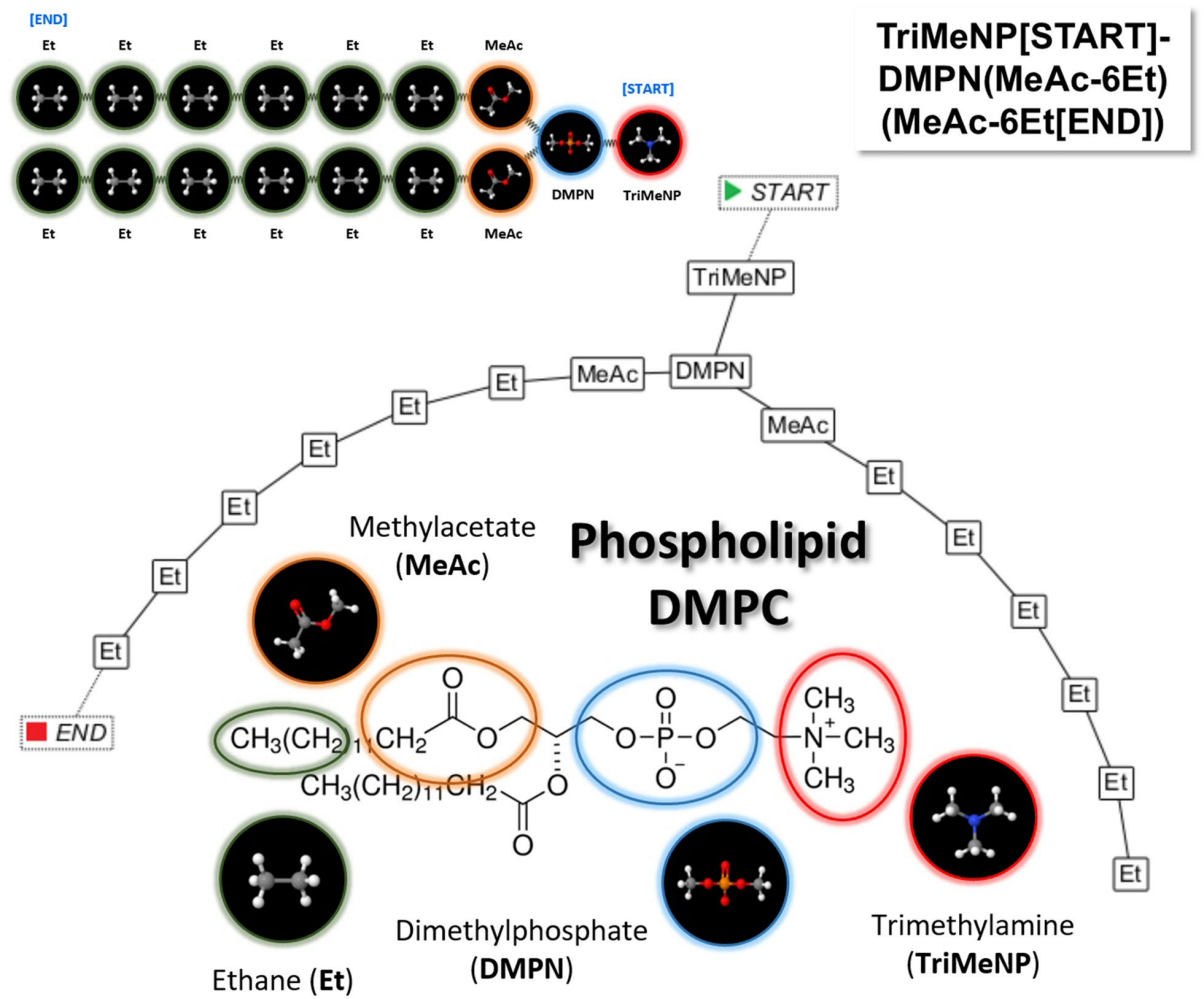
Decomposition of the DMPC phospholipid into “molecular fragment” particles [20] and illustration of the resulting bonded particles (upper left) with corresponding SPICES line notation (upper right): The *SpicesViewer* GUI generated visual particle graph surrounds the “molecular fragment” particle identification

In order to contribute to the realization of a molecular fragment cheminformatics roadmap [9] this work tries to alleviate molecular structure handling and encoding for particle-based mesoscopic simulation techniques like Dissipative Particle Dynamics (DPD) [10–14]: These techniques aim at describing supramolecular phenomena at the nanometer (length) and microsecond (time) scale for large interacting physical ensembles representing millions of atoms. DPD particles in particular may be identified with distinct small molecules of molar mass in the order of 100 Da where larger molecules are composed of adequate “molecular fragment” particles that are bonded by harmonic springs to mimic covalent connectivities and spatial 3D conformations [9, 14–20]. Since no unique molecular fragmentation scheme exists for the various mesoscopic simulation approaches there is nothing like a universal particle set. An adequate decomposition of a chemical compound into appropriate “molecular fragment” particles is a kind of artisan craftwork which is guided by experience, empirical rules and field of application. Figure 2 demonstrates a possible fragmentation for a DMPC phospholipid that successfully preserves its amphiphilic characteristics [20].

Key part of this work is a set of methods operating on an intuitive line notation for particle-decomposed molecular structures denoted SPICES (Simplified Particle Input ConnEction Specification). The SPICES design is derived from straightforward simplifications of the well-established SMILES representation for atom-based molecular connectivity [21–23]. The set of SPICES related methods supports the interplay of structural encoding levels (compare Fig. 1) as well as structure-based calculations for mesoscopic simulations (length and time scales, simulation box size, compound concentrations etc.): It allows for parsing and (graphically) analyzing the line notations, topological calculations (e.g. particle frequencies, particle neighbors or particle paths) as well as the generation of corresponding 3D particle structures with support of their spatial mapping into the simulation box and the final output of tabular ASCII files with molecular information for the following simulation step (the construction of the tabular ASCII file at [1] was in fact supported by the SPICES related code of this work).

## Concept, feature overview and implementation details

**Fig. 3:**
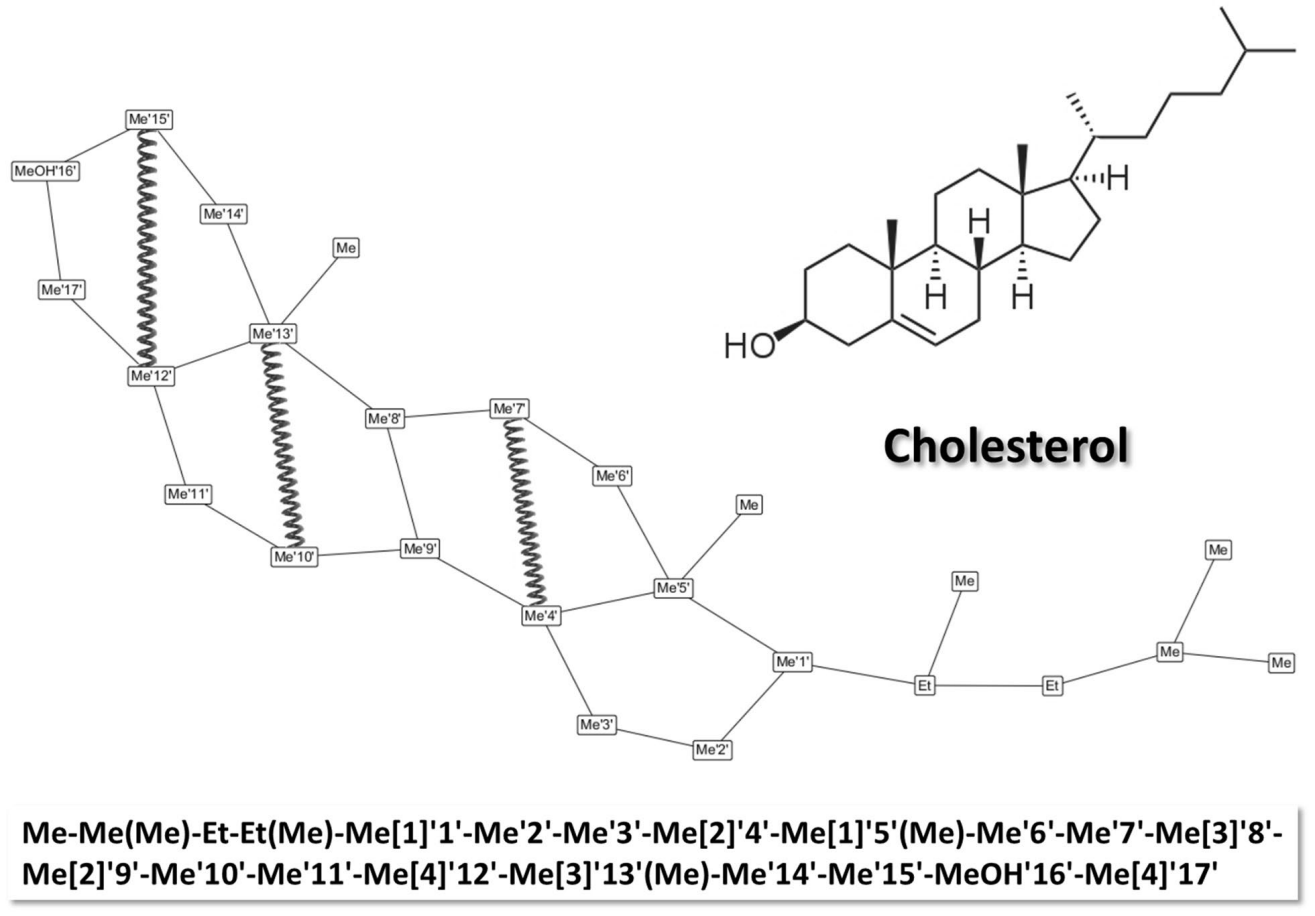
Cholesterol fragmentation scheme with SPICES line notation (at the bottom). The specified backbone labels ‘1’ to ‘17’ allow for an assignment of specific inter-particle forces (e.g. the exemplarily shown harmonic springs between particles Me’12’ and Me’15’, Me’10’ and Me’13’ and Me’4’ and Me’7’) in order to control the stiffness of molecular structure elements like the cholesterol ring structure

The SPICES implementation extends the fragment structure representation proposal in [9]. The syntax rules for a correct SPICES line notation together with some helpful comments are outlined in the appendix. These rules allow arbitrary topological particle connections with branches and ring closures but do not comprise attributes like electric charges or chiral centers since these are intrinsic particle properties (i.e. differently charged states or different enantiomers of a “molecular fragment” particle have to be coded with different particles where each particle has a specific charge and a specific stereochemistry). Particles may possess a “backbone” label which may be utilized to assign specific particle pair forces e.g. for spatial 3D structure constraints of ring structures (see Fig. 3), the tail stiffness of surfactants and lipids or the backbone conformation of macromolecules like proteins. This kind of labeling could be performed in an automated manner by attaching a tagging label to every particle (which in fact was our first approach) but according to our findings the user control of the “backbone” label distribution within a molecule alleviated possible manual force assignments as well as the interplay between the textual line notation and the corresponding visual particle graph. In addition the concrete force assignments are chosen to be not a part of the line notation itself due to their intrinsic differences (from simple springs to e.g. complicated polygonal force chains) and possible automated conditional assignments according to various criteria. Thus the manual “backbone” labels allow for a flexible post-processing for different purposes in the aftermath of molecular definitions.

**Fig. 4:**
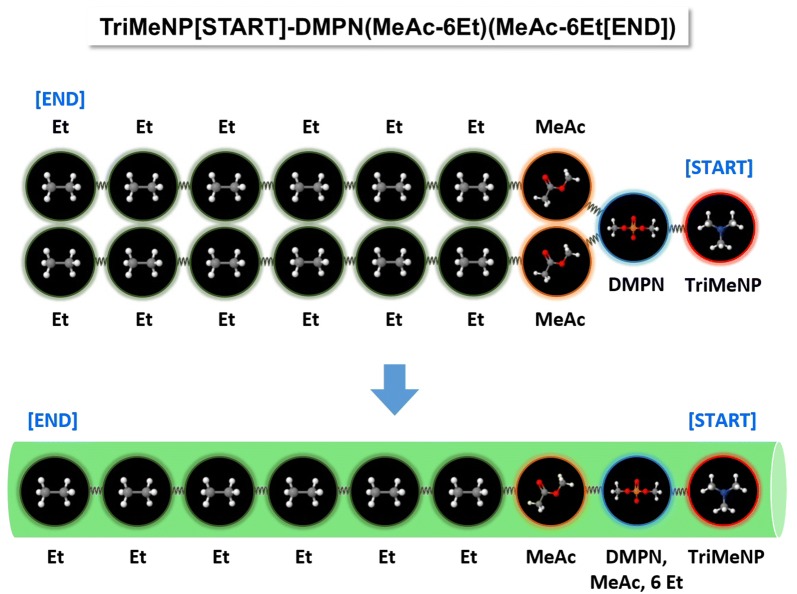
Top: Phospholipid DMPC fragmentation scheme [20] with 16 particles connected by harmonic springs (compare Fig. 2). Bottom: For spatial mapping into the simulation box the topological DMPC particle structure is converted to a linear 3D tube along the [START]/[END] tagged main chain where side-chain particles are collapsed onto the spatial positions of their neighbored main-chain particles, i.e. the second spatial position to the right contains 8 particles with the exact same position: The main-chain particle DMPN and the side-chain particles MeAc and 6 Et

**Fig. 5:**
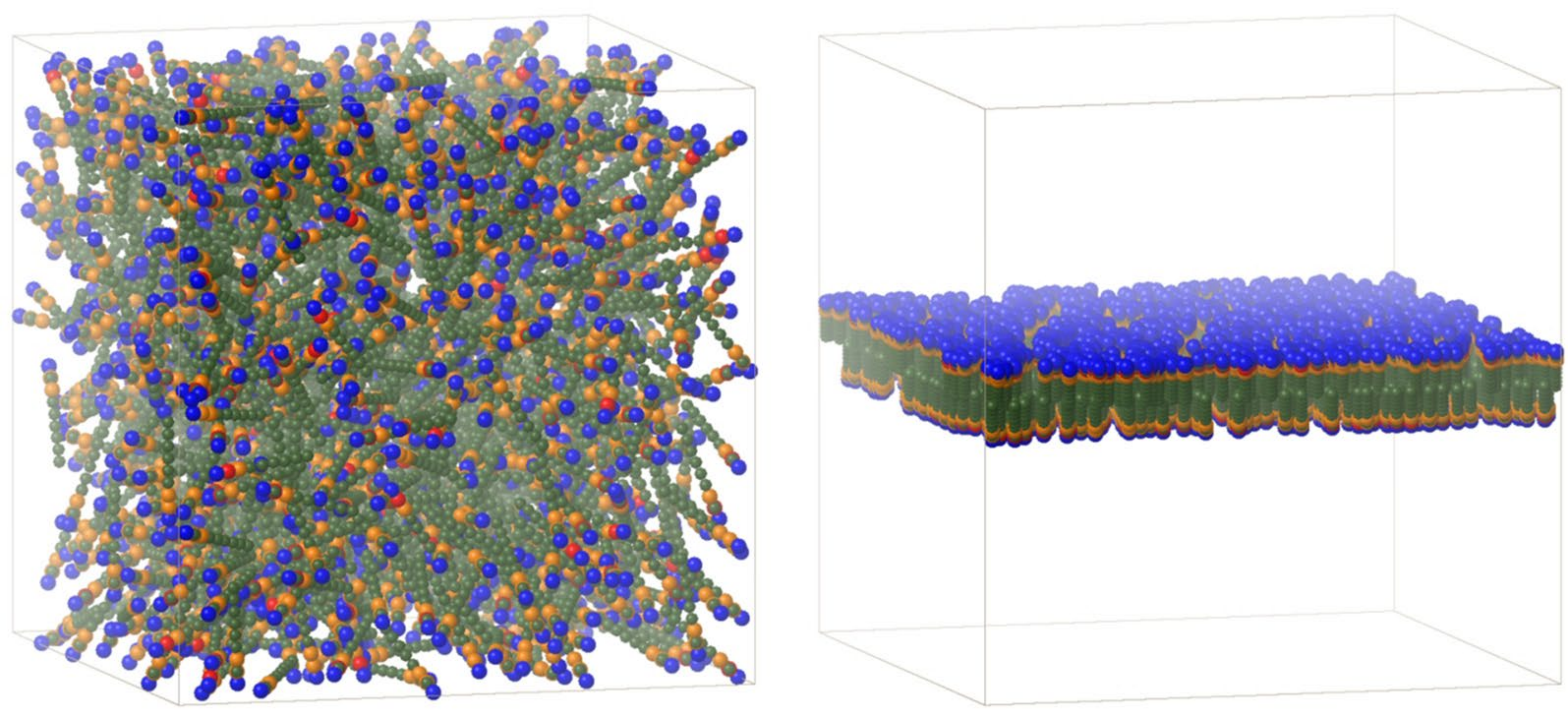
Simulation box start geometry with random distribution (left) or bilayer orientation (right) of phospholipid DMPC molecules as linear 3D tubes (see Figs. 1, 2 and 4). Color code of particles: Et (olive), MeAc (orange), DMPN (red), TriMeNP (blue)

A SPICES representation may contain multiple independent parts (with each part being a valid molecule), e.g. to represent aggregated molecular structures like the quaternary structure of proteins. Finally a [START] and an [END] tag may be attributed for spatial orientation in the simulation box, see Figs. 2, 4 and 5.

The *Spices.jar* library supports all aspects of SPICES definition and handling. A *Spices* object may be created with at least an input structure string or in combination with additional information like a map of available particles. A syntax parser analyzes the provided line notation and returns detailed syntax error information if necessary by the methods *isValid* and *getErrorMessage*. SPICES properties like the frequency of particles or complete lists of particle neighbors are evaluated upon user request by the methods *getParticleFrequencies* or *getNextNeighbors*.

A function of specific importance is the spatial projection of topological SPICES into a simulation box to set up adequate start geometries. Since a mesoscopic simulation is driven by soft particle potentials (in contrast to atomic hard core repulsions for e.g. molecular dynamics), different particles may occupy the same exact spatial position (which would lead to infinite forces for hard atomic potentials) as well as penetrate each other. Thus the possibly severe problems of particle entanglements or caging effects due to inadequate start geometries are considerably attenuated [24]. Nonetheless, a more favorable initial configuration may considerably reduce the necessary simulation period. A straightforward approach is a spatial linear tube representation [9] as shown in Figs. 4 and 5: The longest linear particle chain in the molecule is determined and its particles are consecutively lined up along a straight line according to the specified bond length (which may be squeezed to fit into specific compartments like simulation box layers, see below and Fig. 5). Then all branched side particles are collapsed onto their nearest-neighbor particle on this line. For a fast determination of a sufficiently long linear particle chain, the Depth-First Search (DFS) algorithm is used [25]. Starting from the first particle of the SPICES line notation the maximum-distant particle A is evaluated by a first DFS run. With a second DFS run, the maximum-distant particle B from particle A is determined. Finally the particle chain between A and B is chosen for the spatial tube representation. If a [START]/[END] tag pair is defined the longest (oriented) linear chain between the tagged particles is evaluated. The sketched algorithm leads to true longest chains for acyclic SPICES but not necessarily for cyclic particle structures. For a distinct fragmentation scheme of a molecule there may be several different but equally valid SPICES line notations since the proposed line notation is not canonically unique. For acyclic SPICES with a defined [START]/[END] tag pair the sketched 3D tube construction process will lead to a single distinct spatial 3D tube representation for all these possible different line notations (without a defined [START]/[END] tag pair there may be two possible orientations). For cyclic particle structures this may not be the case, i.e. different but equally valid SPICES line notations may lead to different spatial 3D tube representations and corresponding different start geometries of a simulation. According to our experience this shortcoming is of minor practical relevance since the possibly different 3D tube representations for small molecules seem to be sufficiently similar for convergent mesoscopic simulation results. On the other hand, for large complex molecules like cross-linked (bio)polymers the simple linear 3D tube representation is questionable in principal so that specific conversion tools like a PDB-to-SPICES parser for peptides and proteins would be advised which would take the known molecular 3D structure into account.

The sketched spatial projection (see Fig. 5) is accomplished by interplay of the methods *setCoordinates* and *getParticlePositionsAndConnections*: After creation of a *Spices* object from a SPICES line notation string (which is rapidly performed within a fraction of a second for small molecules like DMPC) arrays for the first (start) and the last (end) particle positions of all spatial linear 3D tubes as well as the bond length may be provided via the *setCoordinates* method. The first (start) particles of the linear chains always have the defined start positions whereas the last (end) particles may not necessarily reach the defined end positions if the length of the defined start/end straight line is longer than the accumulated bond lengths of the particles on the longest linear chain so that a 3D tube may be smaller than defined. On the other hand a 3D tube may be squeezed (with equally reduced bond lengths) if the length of the defined start/end straight line is smaller than the accumulated bond lengths. Thus the calling code (e.g. a compartment editor that allows for flexible compartment definitions within the simulation box like the bilayer compartment shown right in Fig. 5) must only define correctly-oriented and valid lines within an arbitrary compartment (which is comparatively simple to realize) without the necessity to calculate and pre-check every individual length (which could be more difficult). Method *getParticlePositionsAndConnections* then provides all corresponding particle positions within the simulation box where in addition all particle–particle bonds are coded with specific offsets which are commonly used by simulation kernels (compare to the tabular ASCII file at [1]). The sketched interplay of methods *setCoordinates* and *getParticlePositionsAndConnections* performs sufficiently fast for true on-the-fly calculations, e.g. a spatial projection of 50.000 DMPC molecules (with 800.000 particles) into the simulation box performs in less than a second using an ordinary scientific workstation or even a standard notebook computer.

**Fig. 6:**
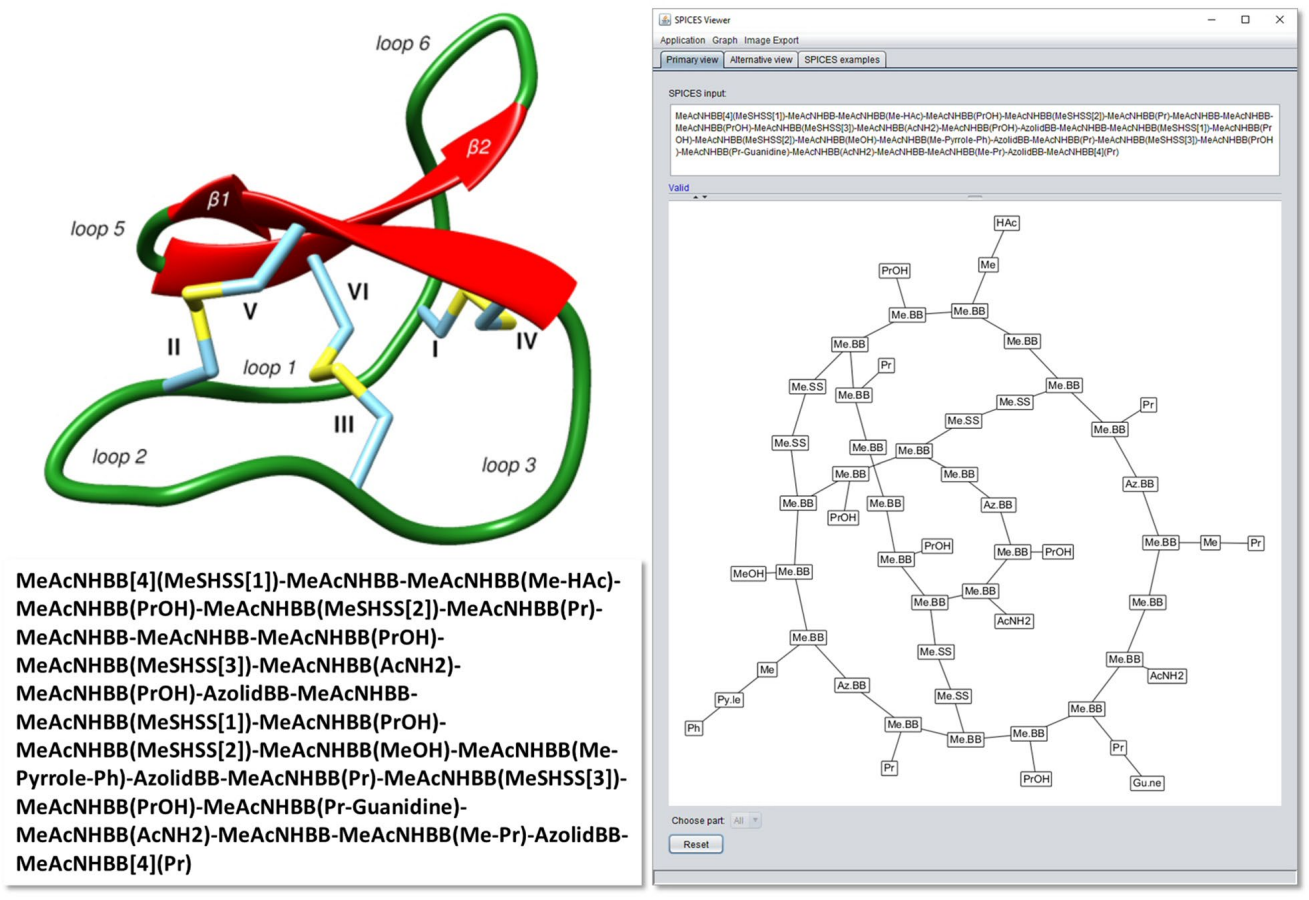
*SpicesViewer* graph display (right) of the cyclotide Kalata B1 (upper left) with 29 amino acids according to the fragmentation scheme in [20] (lower left)

Whereas line notations may be regarded as a reasonable compromise for a human–machine interface (readable by human beings, decomposable by machine) their definitions are error-prone for complex branched or ring structures. A visual display of the topological particle graph with all its particle–particle connections may considerably alleviate a correct SPICES definition, see Fig. 6.

A graphical visualization may be achieved by adequate application of open-source projects that provide chemical structure drawing capabilities. For instance the structure-diagram layout of the Chemistry Development Kit (CDK) [26–28] can be customized to display SPICES instead of atom-based connection topologies [9]. A principle problem of this (mis)use of atom-based layouts is the inappropriateness of its layout elements and templates: Particle graphs do not follow common patterns of atomic connections (see Fig. 6) so that topological visualizations may result in incomprehensible graphs. Thus a more general graph visualization approach with e.g. the *GraphStream* library [29] is necessary. In addition this library allows individually tailored changes of the produced graph by manual displacement of node positions to remove unwanted node or edge overlaps. *SpicesViewer.jar* is a GUI application (on top of *Spices.jar* and connection library *SpicesToGraphStream.jar*) for a topological SPICES display with the *GraphStream* library to analyze the influence of different graph settings and to demonstrate computational functions like zooming or graph image generation. Figure 6 shows the *SpicesViewer.jar* GUI with a manually tailored SPICES graph visualization of the cyclic peptide Kalata B1 with 29 amino acids.

## Conclusions

This work provides a Java library for SPICES handling and mesoscopic simulation support (*Spices.jar*) in combination with a connection library (*SpicesToGraphStream.jar*) and a Java Graphical User Interface (GUI) viewer application (*SpicesViewer.jar*) for visual topological inspection and manipulation of SPICES molecule definitions. All libraries/applications are publicly available as open source published under the GNU General Public License version 3 [30]. The SPICES GitHub repository contains the Java bytecode libraries, a Windows OS installer for the *SpicesViewer* GUI application, all Javadoc HTML documentations [31] and the Netbeans [32] source code packages including Unit tests.

The presented set of methods may alleviate molecular structure definitions for mesoscopic simulation tasks. The *SpicesViewer* GUI application demonstrates relevant use cases in detail with corresponding sample code. The new libraries may be utilized within scripting environments or become part of integrated mesoscopic simulation systems.

Future developments may address SPICES parsers that especially support the more difficult preparation of polymer systems, e.g. a PDB-to-SPICES parser for peptides and proteins provided in form of PDB files (actually, the SPICES string of the Kalata B1 peptide in Fig. 6 was generated from its PDB file with a prototype parser that uses the amino acid fragmentation schemes and connection rules outlined in [20]). Another promising challenge would be a conversion between particle and all-atom representations for an interplay of atomistic and mesoscopic simulation.

## Appendix: SPICES syntax rules

SPICES monomer and molecular structures are constructed according to the following syntax rules:

1. Particle names consist of a maximum of 10 characters (a–z, A–Z, 0–9, the first character is not allowed to be a digit and must be upper case) and an optionally prefixed frequency number.
2. Particle names in molecular structures (but NOT monomers) may be followed by a backbone label (i.e. a number between apostrophe characters, e.g. ‘1’, ‘2’ etc.) for later definition of spring forces between particle pairs where particles must be labeled in a consecutive manner.
3. The connection character ‘–’ is used for bonding between particles.
4. Round brackets ‘(‘ and ‘)’ indicate branches. They may be nested for arbitrary levels of branches.
5. Square brackets ‘[‘ and ‘]’ with an enclosed number which follow a particle indicate a ring closure. Multiple bonds between two particles are counted only once.
6. Curly brackets ‘{‘ and ‘}’ include a monomer definition. Monomers are defined as molecular structures but must contain at least 1 particle with a [HEAD] and [TAIL] attribute: Structure elements that precede the monomer connect to the HEAD particle, structure elements that follow the monomer connect to the TAIL particle. Monomers are not allowed to be nested and backbone labels are forbidden in monomers.
7. Monomer labels start with a ‘#’ character followed by a sequence of characters (first character is not allowed to be a digit and must be upper case).
8. Monomer labels may be preceded by a frequency number (to construct polymers).
9. A molecule may consist of multiple independent parts (i.e. parts are not allowed to be connected in any way). Each part must be framed by angle brackets ‘<‘ and ‘>‘. Parts are not allowed to be nested. Monomers are not allowed to contain parts. A part may have an optional prefixed frequency number.
10. A particle (that is not within a monomer) may optionally contain a [START] or an [END] tag which may be used for orientation purposes. There is only one [START]/[END] pair allowed per independent part.

### Comments

- “A–B–C” defines a connection of particle A with particle B and particle B with particle C.
- “3A–B” is a shortcut notation for “A–A–A–B”.
- “A–2B(E–F)–D” is identical to “A–B–B(E–F)–D”, “3A(B)–D” is a shortcut for “A–A–A(B)–D”. The shorter initial string should be preferred in both cases.
- “A–B(D–E)–F” defines a main chain “A–B–F” with a side chain “D–E” where particle D is connected to particle B.
- “A–B[1]–C–C–C–D–E[1]” defines a ring closure between particles B and E.
- “A[1]–B[1]” is equal to “A–B”, “A[1][2]–B–C–D[1][2]” is equal to “A[1]–B–C–D[1]”: Multiple bonds between two particles are counted only once.
- “A–B(D–E(G–H[1])–F)–I–A–K[1]–B” defines a main chain “A–B–I–A–K–B” with a side chain “D–E–F” (connected to particle B of the main chain) and another side chain “G–H” (connected to particle E of the first side chain). In addition there is a ring closure between particle H of the second side chain and particle K of the main chain.
- “3A[1]–B–B–C[1]” is a shortcut for “A–A–A[1]–B–B–C[1]”.
- “A’1’–B–C–D–E’2’” has two backbone particles. Note that backbone particles must be labeled in a consecutive manner, i.e. “A’1’–B–C–D–E’3’” or “A’1’–B–C–D–E’1’” are forbidden, but “A’1’–B–C’3’–D–E’2’” is valid.
- The shorter (preferred) string “3A’1’–B–C–D–E’2’” is identical to “A–A–A’1’–B–C–D–E’2’”.
- Multiple ring closures at one particle are marked by successive use of ring–closure brackets, e.g. particle B in “A–B[1][2]–4C–D[1]–4C–E[2]” is connected to particles D and E.
- The simplest structure of a monomer consists of a single particle A with attributes [HEAD] and [TAIL], i.e. “{A[HEAD][TAIL]}”.
- In “E–#MyMonomer–F” with #MyMonomer equal to “{A[HEAD]–B–C[TAIL]–D}” particle E is connected to particle A (the head) of the monomer and particle C (the tail) of the monomer is connected to particle F. This definition is equivalent to “E–A–B–C(D)–F”.
- “2{A[HEAD]–B–C[TAIL]–D}” defines a structure of 2 monomers where particle A (the head) of the second monomer is connected to particle C (the tail) of the preceding first monomer. This definition is equivalent to “A–B–C(D)–A–B–C–D”.
- Definitions like “{A[HEAD]–{A[HEAD]–B–B[TAIL]–C}–B[TAIL]–C}” with nested monomers are forbidden.
- “A[START]–B–C[END]” defines orientation information.
- “A[START][END]–B–C” is syntactically correct but makes no sense.
- “A[START]–B[START]–C[END]” is forbidden: There is only one [START]/[END] pair allowed per structure.
- The shorter (preferred) string “3A[START]–B–C[END]” is identical to “A–A–A[START]–B–C[END]”.
- “<A–B–C> <A–D>“ defines a molecule which consists of two independent parts “A–B–C” and “A–D”.
- “<A–B[1]–C> <A–D[1]>“ is forbidden since parts are not allowed to be connected in any way. The correct definition in this case would be “(A–B[1]–C)(A–D[1])” or “A–B(C)–D–A”.
- “3<A–B>“ is equal to “<A–B> <A–B> <A–B>“.
